# cGMP-Dependent Protein Kinase Contributes to Hydrogen Sulfide-Stimulated Vasorelaxation

**DOI:** 10.1371/journal.pone.0053319

**Published:** 2012-12-28

**Authors:** Mariarosaria Bucci, Andreas Papapetropoulos, Valentina Vellecco, Zongmin Zhou, Altaany Zaid, Panagiotis Giannogonas, Anna Cantalupo, Sandeep Dhayade, Katia P. Karalis, Rui Wang, Robert Feil, Giuseppe Cirino

**Affiliations:** 1 Department of Experimental Pharmacology, Faculty of Pharmacy, University of Naples–Federico II, Naples, Italy; 2 Department of Pharmacy, Laboratory of Molecular Pharmacology, University of Patras, Patras, Greece; 3 Developmental Biology Section, Center for Basic Research, Biomedical Research Foundation of the Academy of Athens, Athens, Greece; 4 “G.P. Livanos” Laboratory, First Department of Critical Care and Pulmonary Services, University of Athens School of Medicine, Athens, Greece; 5 Department of Biology, Lakehead University, Thunder Bay, Ontario, Canada; 6 Interfakultäres Institut für Biochemie, Universität Tübingen, Tübingen, Germany; Maastricht University, The Netherlands

## Abstract

A growing body of evidence suggests that hydrogen sulfide (H_2_S) is a signaling molecule in mammalian cells. In the cardiovascular system, H_2_S enhances vasodilation and angiogenesis. H_2_S-induced vasodilation is hypothesized to occur through ATP-sensitive potassium channels (K_ATP_); however, we recently demonstrated that it also increases cGMP levels in tissues. Herein, we studied the involvement of cGMP-dependent protein kinase-I in H_2_S-induced vasorelaxation. The effect of H_2_S on vessel tone was studied in phenylephrine-contracted aortic rings with or without endothelium. cGMP levels were determined in cultured cells or isolated vessel by enzyme immunoassay. Pretreatment of aortic rings with sildenafil attenuated NaHS-induced relaxation, confirming previous findings that H_2_S is a phosphodiesterase inhibitor. In addition, vascular tissue levels of cGMP in cystathionine gamma lyase knockouts were lower than those in wild-type control mice. Treatment of aortic rings with NaHS, a fast releasing H_2_S donor, enhanced phosphorylation of vasodilator-stimulated phosphoprotein in a time-dependent manner, suggesting that cGMP-dependent protein kinase (PKG) is activated after exposure to H_2_S. Incubation of aortic rings with a PKG-I inhibitor (DT-2) attenuated NaHS-stimulated relaxation. Interestingly, vasodilatory responses to a slowly releasing H_2_S donor (GYY 4137) were unaffected by DT-2, suggesting that this donor dilates mouse aorta through PKG-independent pathways. Dilatory responses to NaHS and L-cysteine (a substrate for H_2_S production) were reduced in vessels of PKG-I knockout mice (PKG-I−/−). Moreover, glibenclamide inhibited NaHS-induced vasorelaxation in vessels from wild-type animals, but not PKG-I−/−, suggesting that there is a cross-talk between K_ATP_ and PKG. Our results confirm the role of cGMP in the vascular responses to NaHS and demonstrate that genetic deletion of PKG-I attenuates NaHS and L-cysteine-stimulated vasodilation.

## Introduction

Hydrogen sulfide is a small gaseous compound that together with nitric oxide and carbon monoxide comprises the gasotransmitter family [1], [2]. Initially viewed as environmental pollutants and biohazardous compounds gasotransmitters are now widely accepted for their important roles in physiology and disease [3], [4], [5], [6]. Hydrogen sulfide is the newest and least studied gasotransmitter. However, recently there has been a surge of interest in hydrogen sulfide biology leading to important observations regarding its role in mammalian cells. H_2_S has been proposed to modulate inflammatory responses, participate in neurotransmission and affect smooth muscle and heart function [7], [8]. In the body, hydrogen sulfide is produced by both enzymatic and non-enzymatic sources. The enzymes implicated in H_2_S generation include cystathionine beta synthase (CBS), cystathionine gamma lyase (CSE) and 3-mercaptopyruvate sulfurtransferase (3MST) [5], [9]. It is believed that CSE is the primary source of H_2_S in the vasculature, while CBS exists in higher levels in the nervous system [8]. While 3MST has been shown to be present in endothelial cells [10], this enzyme is relatively less studied and its role in cardiovascular biology is unclear.

Hydrogen sulfide has been shown to exhibit a variety of biological effects in the cardiovascular system. It exerts anti-apoptotic and cardioprotective effects in cardiomyocytes, stimulates the angiogenic properties of endothelial cells and alters vessel tone [6], [11], [12], [13]. Although constrictor effects have been observed in response to H_2_S, H_2_S is mostly viewed as a vasorelaxing agent [11], [14], [15]. The antihypertensive role of endogenously produced H_2_S is corroborated by observations that pharmacological inhibition of H_2_S production [16], [17], [18], as well as targeted disruption of the CSE locus leads to an increase in blood pressure in animals [19]. Moreover, administration of H_2_S reduces mean arterial blood pressure and causes vasorelaxation of conduit and resistance vessels [11], [19], [20], [21]. Several mechanisms have been proposed to contribute to the effects of H_2_S on vessel tone. Initially, H_2_S was shown to enhance vasorelaxation by promoting K_ATP_ channel opening [21]. However, additional pathways contribute to vasorelaxation in response to H_2_S, as K_ATP_ channel blockers fail to inhibit or do not completely abolish H_2_S-induced relaxations in some tissues [15], [22]. These additional vasodilatory pathways might include other ion channels, as well as cGMP-nucleotide regulated pathways [15], [22]. With respect to the latter, we have recently observed that H_2_S increases cGMP levels in smooth muscle cells [23]. Unlike nitric oxide (NO) that enhances cGMP synthesis by activating soluble guanylyl cyclase, elevations in cGMP in response to H_2_S result from phosphodiesterase (PDE) inhibition. The aim of the present study was to further analyze the role of cGMP in H_2_S-stimulated vasorelaxation and to determine the contribution of cGMP-dependent protein kinase in H_2_S responses.

## Results

### PDE regulates H_2_S-induced relaxation

**Figure 1:**
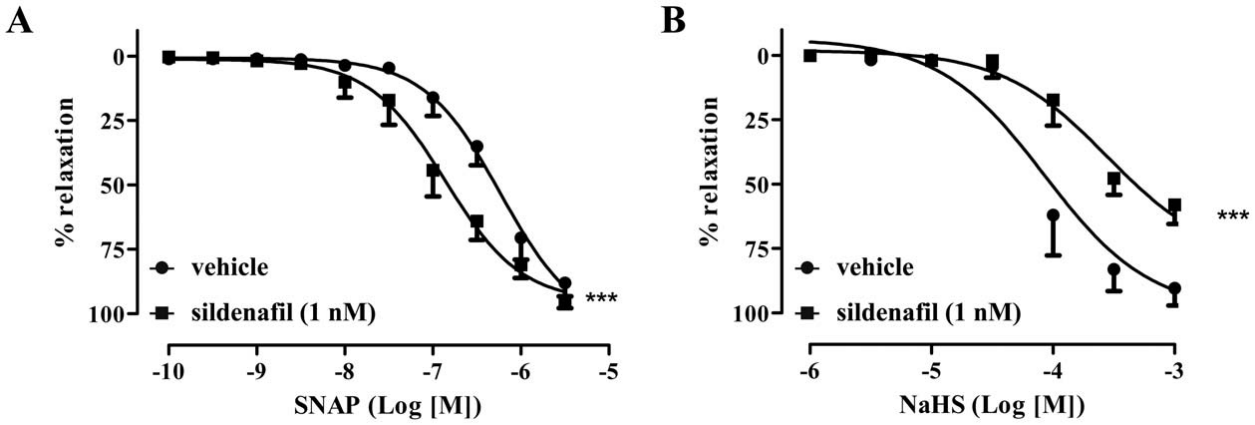
The PDE5 inhibitor sildenafil differentially affects NO and H_2_S-regulated vascular tone. (A) Incubation of isolated aortic rings with sildenafil (1 nM) significantly inhibited NaHS-induced vasodilatation. (B) Incubation of isolated aortic rings with sildenafil (1 nM) significantly enhanced SNAP-induced vasodilatation. *** p<0.001 vs vehicle (dH_2_O), n = 6 for each group.

We have previously demonstrated that exposure of smooth muscle cells to NaHS increases cGMP by inhibiting PDE [23]. To test whether our biochemical observations are functionally relevant, we pre-incubated rat aortic rings with a low concentration of the PDE5 inhibitor sildenafil (1 nM) and then contracted them with phenylephrine. Such pre-treatment did not have a significant effect on the ability of phenylephrine to cause tissue contraction, but differentially affected NO-induced vs H_2_S-induced vasorelaxation. Incubation of rings with sildenafil led to a potentiation of NO-induced relaxation as evidenced by the leftward shift of the SNAP dose-response curve (6×10^−7^ M vs. 1.4×10^−7^ M vehicle vs sildenafil, p<0.001; Fig. 1A). In contrast to the findings with the NO donor, pre-treatment with sildenafil attenuated the relaxing effect of NaHS in rat aorta (Fig. 1B). The observed rightward shift of the NaHS dose-response in the presence of sildenafil (2.8×10^−4^ M vs. 8.5×10^−5^ M vehicle vs sildenafil p<0.001) is consistent with the notion that NaHS-stimulated vasodilation is at least in part mediated by PDE5 inhibition.

**Figure 2:**
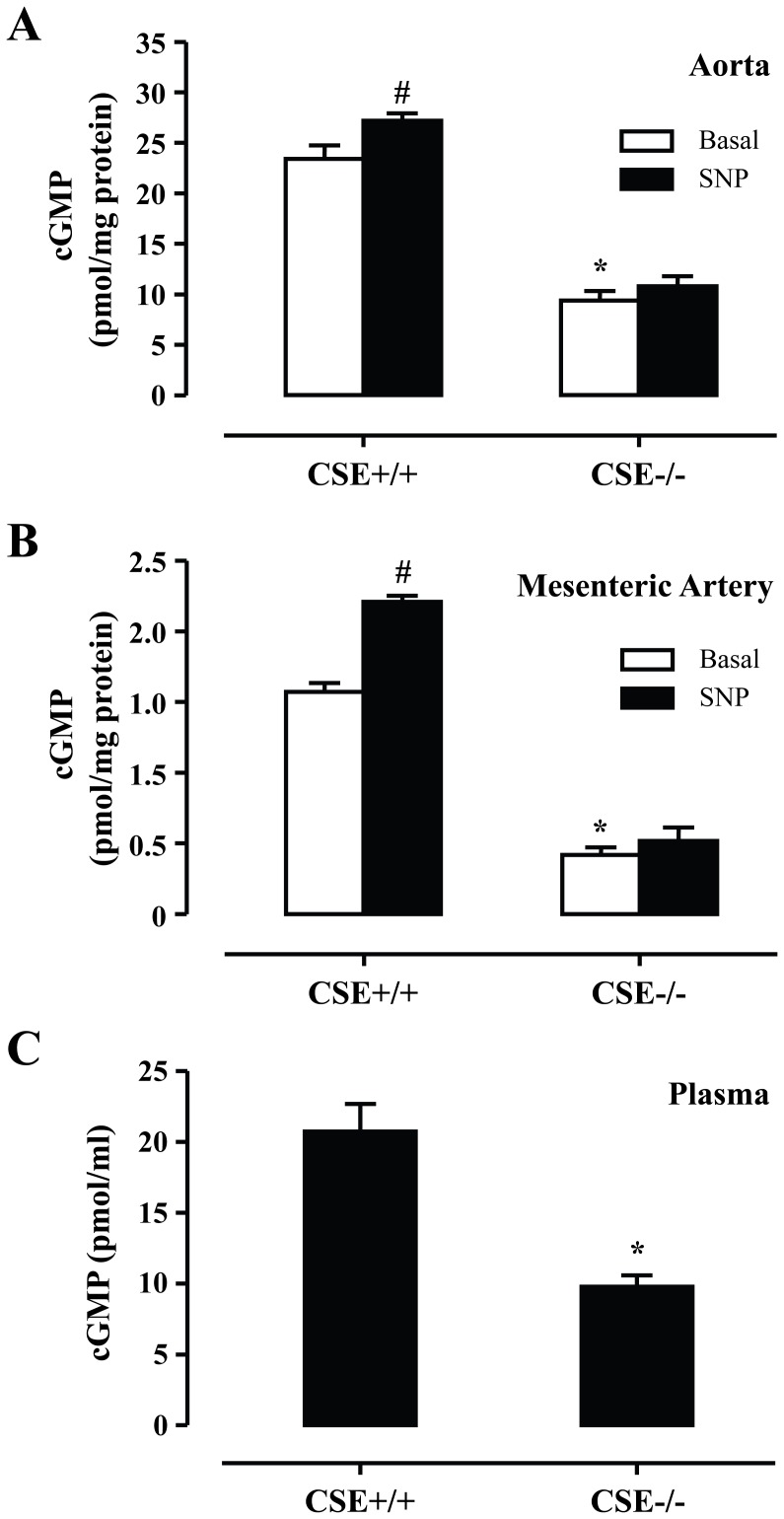
CSE deficiency reduces cGMP levels. cGMP levels in the aorta (A), mesenteric artery (B) and plasma (C) of CSE−/− mice were significantly lower than those from CSE+/+ mice. Sodium nitroprusside (SNP, 10 µM) significantly increased cGMP levels in aorta (B) and mesenteric artery (C) from CSE+/+ mice but not CSE−/− mice; * p<0.05 vs CSE+/+ mice, #p<0.05 basal, n = 5 for each group.

To provide proof that endogenously produced H_2_S acts as a PDE inhibitor, we measured cGMP levels in the plasma and vascular tissues of CSE−/− mice. In these experiments we observed cGMP levels in the plasma (9.81±0.75 pmole/ml), aorta (9.55±0.80 pmole/mg protein), and mesenteric artery (0.42±0.04 pmole/mg protein) from CSE−/− mice were significantly lower than those in the plasma (20.74±1.97 pmole/ml), aorta (23.40±1.44 pmole/mg protein) and mesenteric artery (1.57±0.05 pmole/mg protein) from CSE+/+ mice (Fig. 2). In addition, stimulation of vascular tissues with sodium nitroprusside increased cGMP levels in a statistically significant manner only in the vessels of wild-type, but not in the vessels of CSE−/− animals. The above observations taken together are consistent with the idea that H_2_S is an inhibitor of PDE activity in vascular tissues.

### H_2_S activates PKG in vascular tissues

**Figure 3:**
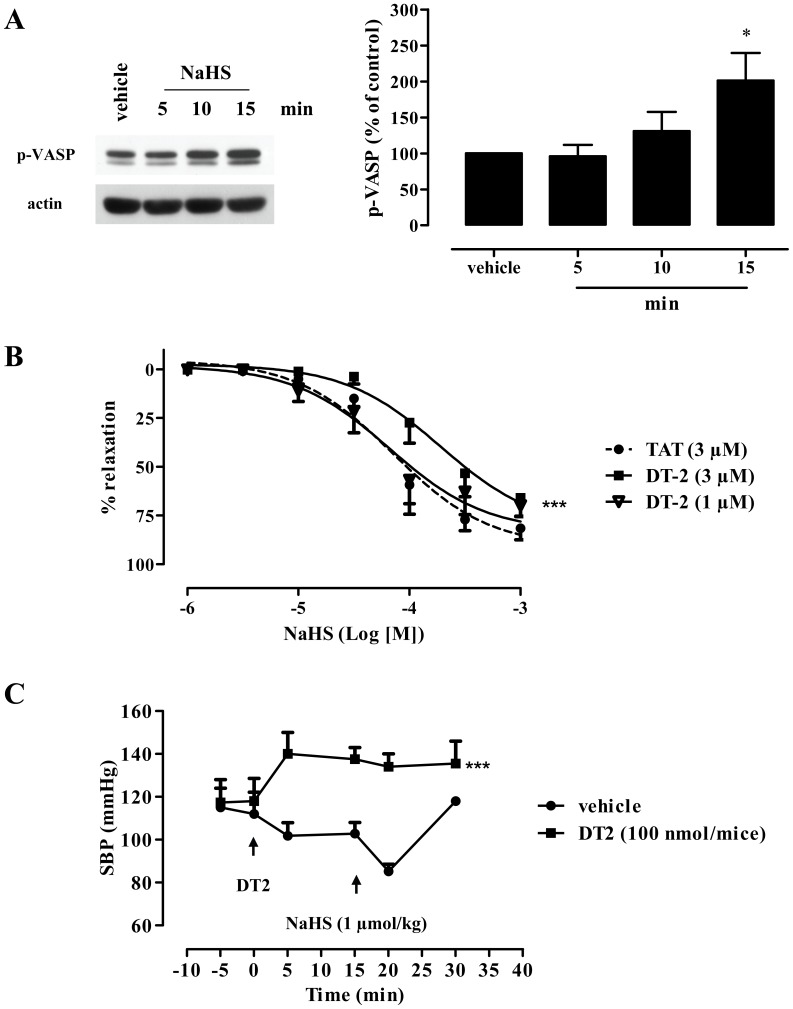
H_2_S activates PKG and triggers vasodilatation. (A) Mouse aorta was incubated with NaHS (50 µM) for the indicated time and VASP phosphorylation on Ser239 was determined. Left: representative blot; right: quantitation of scanned autoradiograms, *p<0.05 vs vehicle, n = 3. (B) Incubation of aortic rings with the selective inhibitor of PKG, DT-2 (1, 3 µM) significantly inhibited NaHS-induced vasodilatation. TAT peptide (3 µM) was used as a control; *** p<0.001 vs. vehicle (dH_2_O), n = 6 for each group. (C) Mice were injected with vehicle or DT-2 (100 nmoles, ip); after 15 min NaHS (1 µmol/kg) was administered subcutaneously. Systolic blood pressure (SBP) was monitored in conscious mice; *** p<0.001 vs vehicle, n = 4 for each group.

To elucidate the downstream signalling pathways activated in response to increased intracellular cGMP, we evaluated the ability of NaHS to stimulate cGMP-dependent protein kinase. To this end, we determined VASP phosphorylation on Ser239, as an index of PKG activation [24]. Indeed, exposure of aortic tissue to NaHS enhanced vasodilator stimulated phosphoprotein (VASP) phosphorylation in a time-dependent manner (Fig. 3A). Moreover, incubation of rings with DT-2, a PKG-I inhibitor, attenuated NaHS-induced vasorelaxation while TAT (control peptide) had no effect (Fig. 3B); these findings provide evidence that PKG-I participates in H_2_S-stimulated dilatation. To study the role of PKG-I in the hypotensive effect of H_2_S *in vivo*, mice we pre-treated with DT-2 prior to being treated with the NaHS (Fig. 3C). DT-2 administration led to an increase in systolic blood pressure (SBP). Subcutaneous injection of NaHS resulted in a fall in SBP that reached a trough, 5 min after the injection, with a complete recovery within 15 minutes. In contrast, NaHS did not alter SBP in mice treated with the PKG-I inhibitor.

**Figure 4:**
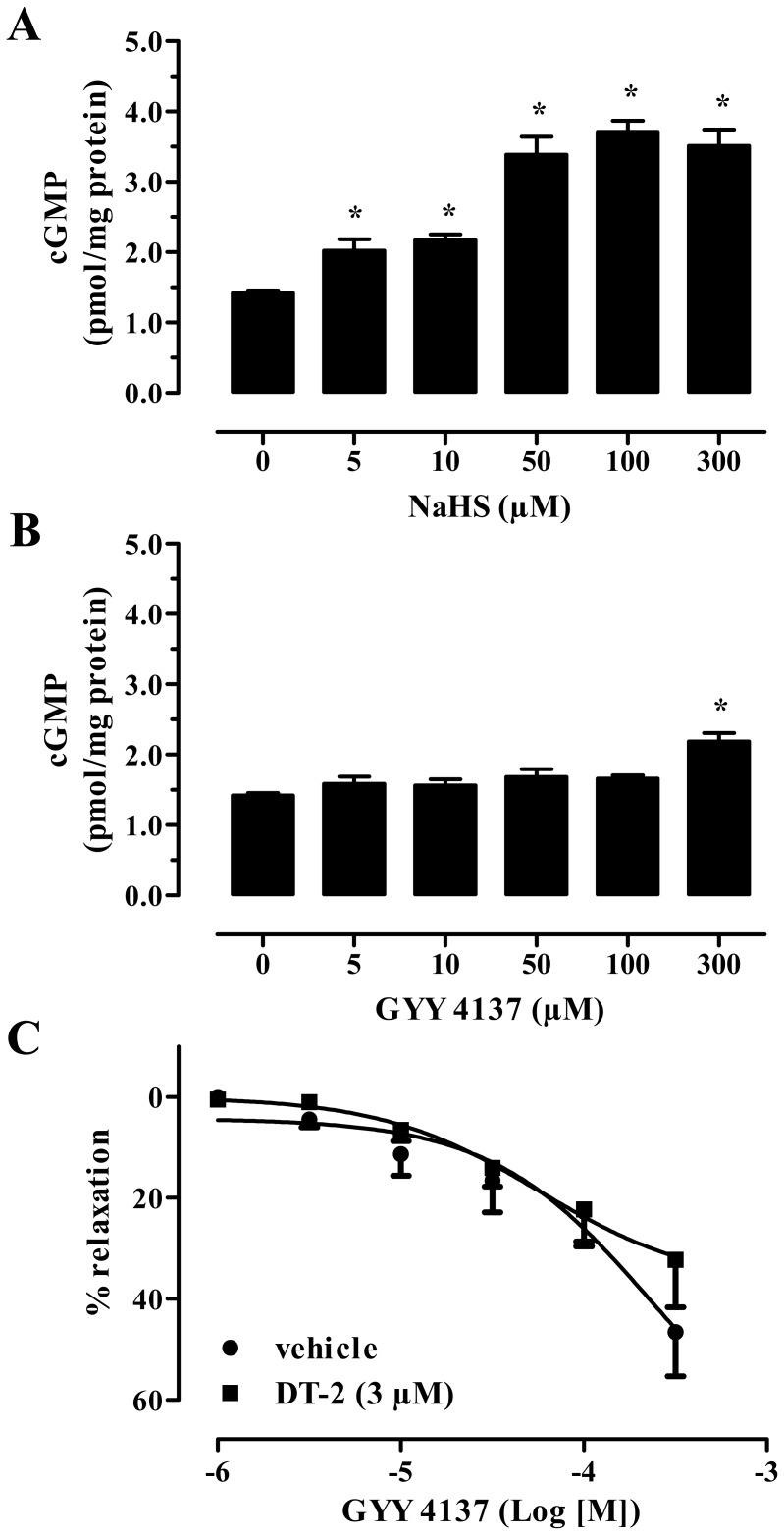
GYY4137-induced relaxation is independent of PKG. Aortic smooth muscle cells were exposed to the indicated concentration of NaHS (A) or GYY4137 (B) and cGMP levels were determined after 5 min. *p<0.05 control, n = 4 for each group. (C) Incubation of isolated aortic rings with PKG selective inhibitor DT-2 (3 µM) did not affect GYY4137-induced vasodilatation; n = 6 for each group.

In a different set of experiments we utilized GYY4137, a slow releasing H_2_S donor. It should be noted that incubation of aortic smooth muscle cells with GYY4137, unlike NaHS, resulted in only minor increases in cGMP content (Fig. 4A&B). In agreement to what has been published, relaxation in response to GYY4137 took longer to manifest compared to the fast relaxations brought about by NaHS [20]. Moreover, GYY-4137-stimulated relaxations were PKG-independent, as DT-2 failed to block the effects of this H_2_S donor (Fig. 4C).

### Genetic evidence for the role of PKG-I in H_2_S-induced vasorelaxation

**Figure 5:**
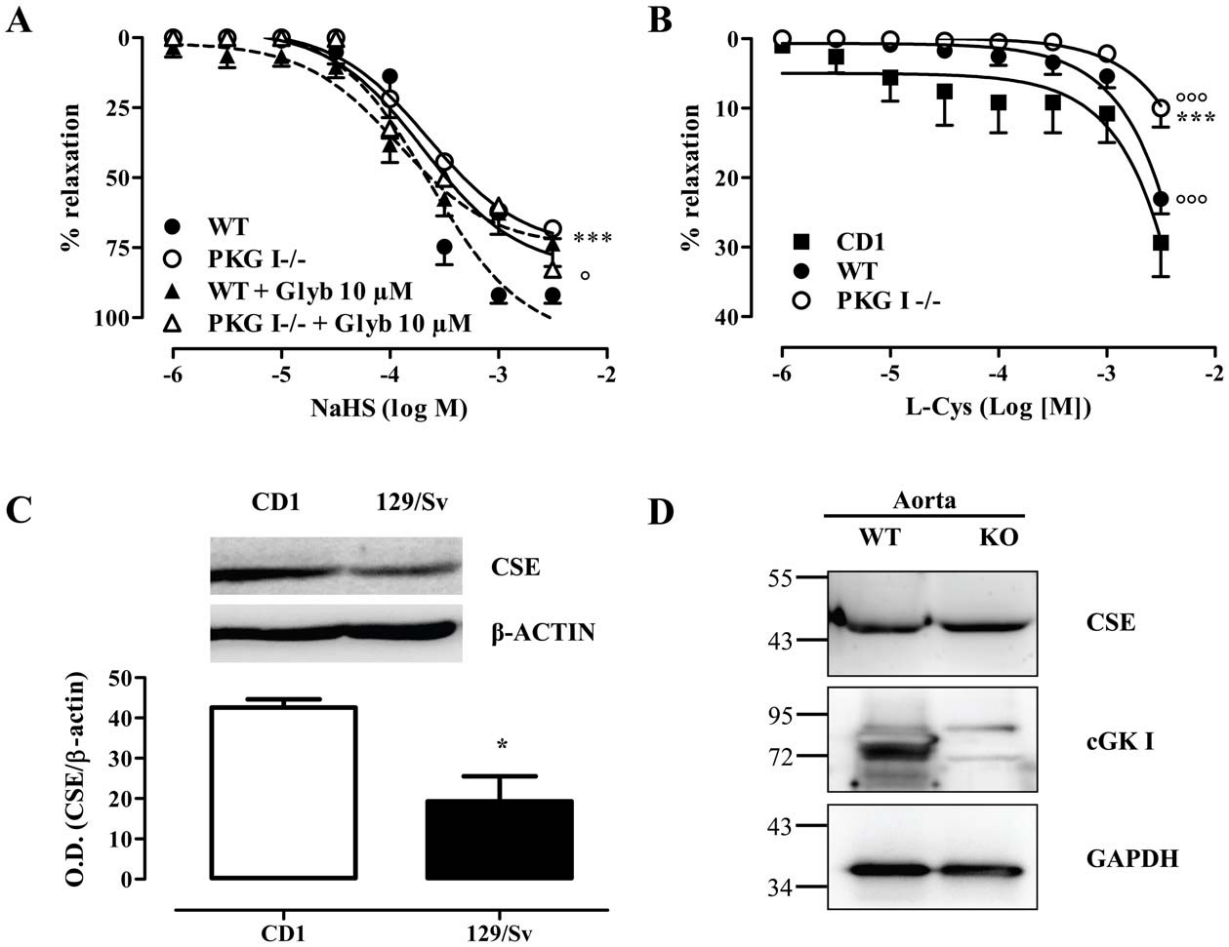
PKG contributes to the relaxing effect of exogenous and endogenous H_2_S. (A) Mouse aortas from wild-type or PKG-I−/− animals were pre-treated with vehicle or glibenclamide (10 µM, 30 min) and then incubated with the indicated concentration of NaHS (n = 6 rings harvested from 3–4 animals); dashed lines are used for wild-type animals, while solid lines are used for knockouts. (B) L-cysteine-induced vasodilatation of aortic rings pre-contracted with phenylephrine from wild-type and PKG-I−/− mice. Note that cumulative concentration-response curves to L-cysteine were significantly different among the different strains of mice used CD1 vs 129/Sv (WT); °°° p<0.001 vs. CD1, *** p<0.001 vs WT, n = 8 rings harvested from 3–4 animals for each group (C) Representative blot and quantitation depicting aortic CSE expression in 129/Sv vs CD-1; n = 3 for each group, *p<0.05. (D) Representative blot showing expression of CSE in aortic homogenates in wild-type and PKG-I−/− mice. Experiments were performed twice with similar results.

Although DT-2 is the most selective PKG-I inhibitor available, questions regarding its specificity have surfaced [25]. To confirm that H_2_S uses PKG-I-regulated pathways to reduce vascular tone, we utilized blood vessels from PKG-I−/− mice. In these experiments we found that relaxations to NaHS were significantly hampered in PKG-I−/− vessels (Fig. 5A); however a significant residual response was observed, suggesting that complementary vasodilator pathways do exist. Importantly, glibanclamide, a K_ATP_ channel inhibitor, blocked NaHS-induced dilation in vessels from wild-type, but not PKG-I −/− mice, suggesting that PKG-I and K_ATP_ work on the same effector pathway to trigger vasodilation. To study whether PKG-I is important for the dilatation in response to endogenously produced H_2_S, rings were exposed to L-cysteine, the substrate for H_2_S generation, and reduction in vessel tone was measured. L-cysteine promoted vasorelaxation in the vessels of wild-type mice; this response was greatly reduced in PKG-I−/− vessels (Fig. 5B). The diminished relaxation to L-cysteine observed in the PKG-I−/− mice was not due to lower levels of the H_2_S producing enzyme CSE in PKG-I −/− aortic tissue (Fig. 5D). It should be noted that relaxations in response to L-cysteine were of smaller magnitude in the 129/Sv mice (wt mice) compared to those observed in CD1 used in the first series of our experiments (max relaxation 30±4.89 vs. 23±2.14 in CD1 and 129/Sv respectively). This difference can be attributed to the lower levels of CSE expression in the aortas of 129/Sv mice (Fig. 5C).

### H_2_S stimulates relaxations in both endothelium intact and denuded aortic rings

**Figure 6:**
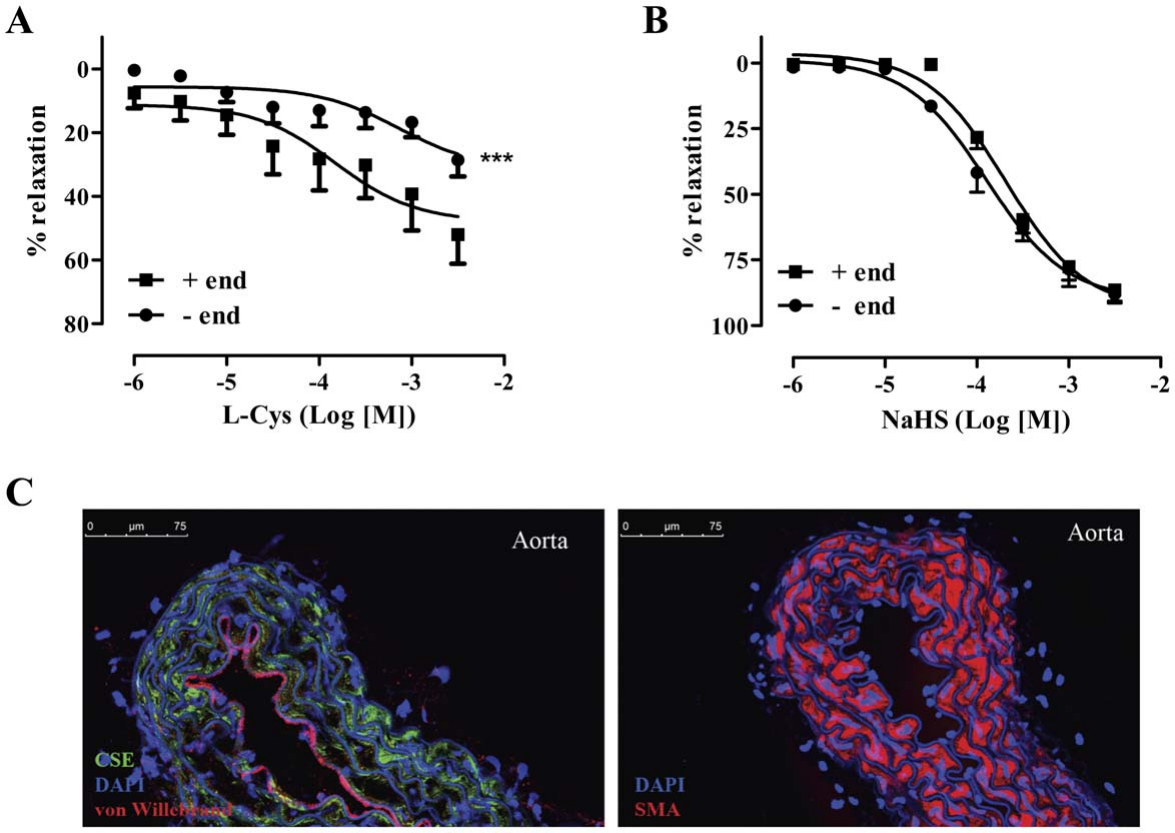
Role of endothelium in H_2_S induced-vasodilatation. (A) L-cysteine-induced vasodilatation was significantly impaired in aortic rings without endothelium (–end). (B) NaHS-induced vasodilatation is not affected by endothelium removal; *** p<0.001 vs –end, n = 6 for each group. (C) Representative photomicrographs of aortas stained with a CSE antibody and counter-stained for von-Willebrant factor, smooth muscle α-actin (SMA) and DAPI, showing localization of CSE.

To test the relative contribution of each cell type (endothelium vs smooth muscle) to the relaxing effect of H_2_S, endothelium intact or endothelium denuded mouse (CD 1) vessels were exposed to either a H_2_S donor or a H_2_S substrate (L-cysteine). While the vasodilatory response to NaHS was identical irrespective of whether endothelium was present or not (Fig. 6B), responses to L-cysteine were reduced in endothelium-denuded vessels (Fig. 6A). Staining of aortic tissue with an antibody against CSE revealed that although small amounts of the enzyme are present in the endothelium, the majority of CSE is expressed in the smooth muscle layer (Fig. 6C).

## Discussion and Conclusions

Relaxation to H_2_S is reported to occur through K_ATP_ channel activation [21], leading to the hypothesis that H_2_S is an endothelium-derived hyperpolarizing factor [11], [26]. The effect of H_2_S on these channels has been proposed to result from sulfhydration of Cys 43 of the pore-forming Kir6.1 subunit and/or interactions with Cys6 and Cys26 of the regulatory subunit SUR1 [26], [27]. Despite the large number of publications proving K_ATP_ channel involvement in the dilatory responses to H_2_S, H_2_S-induced vascular relaxation is only partially inhibited by glibenclamide [6], [15], [21], [28]. There are also instances where K_ATP_ channel inhibition does not attenuate H_2_S-induced vasorelaxation [29], [30]. Based on the ability of H_2_S to increase cGMP levels in vascular tissues, herein we investigated the role of this cyclic nucleotide in H_2_S-induced vasorelaxation and the interaction between cGMP-regulated pathways and K_ATP_ channels in mediating the effects of H_2_S.

As we have previously shown that H_2_S inhibits PDE activity [23], initially, we sought to determine whether H_2_S-triggered relaxation is mediated by inhibition of PDE. Inhibition of PDE5 by sildenafil blocks cGMP breakdown and leads to a reduction in vascular tone [31]. PDE5 blockade has been shown to potentiate the vasodilatory action of NO donors in the aorta [31]. In our experimental setup we confirmed that pre-treatment of mouse aortic rings with sildenafil potentiated the dilatory response to SNAP. In contrast, pre-incubation with sildenafil lead to a rightward shift and reduced the maximal response to NaHS, suggesting that H_2_S, at least in part, relaxes vascular tissue by inhibiting PDE5. To obtain additional evidence that H_2_S regulates cGMP levels we used tissues from CSE−/− mice. Under basal conditions cGMP levels in the aorta and mesenteric artery were lower in CSE−/− compared to wild-type controls. In addition, although a significant increase in cGMP levels was observed after exposure to a NO donor in vessels from wt animals, no such increase was seen in CSE−/− Taken together this data suggest that H_2_S relaxes blood vessels by modulating cGMP levels.

We next sought to determine the signalling pathways downstream of cGMP that become activated after H_2_S exposure and lead to vasodilation. cGMP is known to activate cGMP-dependent protein kinases and to modulate the activity of cGMP-gated ion channels and phosphodiesterases [32]. To evaluate the ability of NaHS to activate PKG we used VASP phosphorylation on Ser239, a site that is preferentially phosphorylated by PKG. Phosphorylation of this VASP residue in vascular extracts is widely used as an index of NO/cGMP pathway activity [24]. Exposure of aortic rings to NaHS resulted in a time-dependent phosphorylation of VASP, suggesting that NaHS activates PKG. Our observations are in line with those of Hu et al. who reported that ischemic myocytes to NaHS exhibited increased PKG activity [33].

To investigate the contribution of PKG to NaHS-induced vasodilation, vessel rings were pre-treated with DT-2 [34] prior to exposure to NaHS. Such pre-treatment attenuated the vasorelaxation brought about by NaHS, indicating that NaHS-induced relaxation is PKG-I-dependent.. One of the mechanisms through which PKG elicits vasorelaxation is activation of myosin phosphatase (PP1M) MLCP, than in turn inhibits MLC phosphorylation [32]. In agreement with our findings, relaxation in response to H_2_S in the mouse gastric fundus is partially blocked by a MLCP inhibitor [35]. So far, studies designed to evaluate the role of cGMP in H_2_S-induced relaxation have reported mostly negative results. In many cases authors were unable to inhibit NaHS-stimulated relaxation by inhibiting soluble guanylyl cyclase activity (sGC) [21], [29], [36], [37]. cGMP levels might still rise in response to H_2_S donors in ODQ-treated tissues, as PDE rather than sGC is the target for H_2_S; cGMP in vascular tissues incubated with ODQ can be synthesized by the basal sGC activity (ODQ does not inhibit basal sGC activity [38]), as well as through natriuretic peptide receptors.

In our next series of experiments we utilized GYY4137, a slow releasing H_2_S donor and determined the contribution of cGMP/PKG pathway to vasorelaxation. In line with previous findings, GYY4137 relaxed pre-contracted aortic rings [20], but elicited a smaller dilatory response with slower kinetics; maximal relaxation to GYY4137 was 46% and took 90–120 min to occur, while NaHS relaxed aortic rings >80% within 30 min. Another striking difference between the two donors was their differential sensitivity to PKG inhibition. Unlike what was observed with NaHS, GYY4137-induced relaxation was not inhibited by DT-2. Also, exposure of smooth muscle cells to GYY4137 at concentrations below 0.3 mM failed to enhance cGMP levels in smooth muscle cells. The difference in the rate of H_2_S release and thus the concentrations of H_2_S achieved after administration of a given dose of each H_2_S donor could explain the difference in their ability to inhibit PDE and enhance cGMP levels in cells. Although it is frequently claimed and intuitively makes sense that slow H_2_S release from donors is more physiologically relevant, endogenous H_2_S production has never been compared to the rate of H_2_S release from this slow H_2_S donor, neither has the half-life of GYY4137 been determined in any biological system, *in vitro* or *in vivo*. The time required for GYY4137 to elicit vasorelaxation, compared to the fast responses triggered by L-cysteine, indicates that it might require bioactivation or that GYY4137 releases H_2_S at a much slower rate than that produced endogenously. In line with the minimal amounts of H_2_S liberated from this donor, when high GYY4137 concentrations are used, PDE inhibition becomes apparent. Thus, one might hypothesize that lower GYY4137 concentrations that trigger sub-maximal vasodilation occur through cGMP-independent pathways (this would represent the 50% residual dilation seen in PKG-I−/− animals after NaHS), while at higher H_2_S concentrations cGMP pathways become important. In addition to the herein presented finding that the fast H_2_S donor NaHS increases cGMP levels in smooth muscle cells, we have demonstrated that slow releasing H_2_S donors (thioglycine, thiovaline) are also capable of increasing cGMP in this cell type [39]. Taken together our findings suggest that researchers utilizing different H_2_S donors with varying half-lives and modes of H_2_S release, should not assume the participation of cGMP/PKG pathways in the observed responses, but should rather determine cGMP levels and PKG activation after application of the donor in their system.

DT-2 is a peptide inhibitor that was originally described as being highly selective for the PKG isoform expressed in vessels, PKG-I [34]. It is 1000-foldmore selective for PKG vs. PKA and exhibits a 100-fold selectivity for PKG-I vs. PKG-II. However, questions regarding the behaviour and specificity of this inhibitor in intact cells have emerged [25]. Gambaryan et al., reported that DT-2 modulated the activity of ERK, p38, PKB and PKC. To prove the involvement of PKG-I in H_2_S-induced vasodilation we used a genetic model. Mice with targeted disruption of the PKG-I locus exhibited a reduced maximal relaxing response to NaHS. To test if endogenously produced H_2_S also reduces vessel tone through PKG-I activation, vessels were exposed to L-cysteine and vascular tone determined. Similarly to what was observed with NaHS, relaxations to L-cysteine were reduced in the PKG-I−/− animals, providing genetic evidence that relaxation in response to both exogenously applied H_2_S (NaHS) and endogenously produced H_2_S are mediated in part through PKG-I. Moreover, inhibition of K_ATP_ channels with glibenclamide led to an inhibition of NaHS-induced vasorelaxation in rings from wild-type mice, that was of similar magnitude to that observed in PKG-I−/− animals. It should be emphasized that glibenclamide did not exhibit an additional inhibitory effect on NaHS dilations in PKG-I−/− mice, suggesting that K_ATP_ and PKG-I work in tandem to promote vasorelaxation. Evidence that PKG activates K_ATP_ channels in the cardiovascular system has been previously reported [40], [41], [42], [43]. It should however be noted that a substantial relaxation (approximately 50%) was still observed in the vessels of PKG−/− mice, providing proof that additional pathways become activated by NaHS and allow H_2_S to reduce vessel tone. The K_ATP_-insensitive dilatory response to NaHS might occur through voltage-dependent K^+^ channels [15], and intracellular acidification through activation of Cl-/HCO_3_^−^ [33]. The relative contribution of cGMP/PKG pathways vs alternative pathways in H_2_S vasorelaxation are expected to vary with the vascular bed and species studied. In addition to the relaxing effect of NaHS on pre-contracted rings, we also observed that NaHS administration *in vivo* reduced systolic blood pressure in a DT-2 sensitive manner. However, since the requirement for PKG-I in the drop in blood pressure elicited by NaHS was only shown using a pharmacological inhibitor for which concerns have been raised [25], ultimate proof that PKG-I mediates the reduction in mean arterial blood pressure triggered by H_2_S will have to await conformation by a genetic model.

In the course of our experiments we noticed that L-cysteine exerted a somewhat smaller effect in the aortic rings of the control mice (wt) compared to the dilation we routinely get in response to this H_2_S synthesis substrate. As the PKG-I−/− mice have been generated on a 129/Sv genetic background, we compared L-cysteine-induced relaxation in CD-1 and 129/Sv mice. Indeed, we observed that L-cysteine-induced relaxations were attenuated in 129/Sv mice compared to CD-1 and this reduced response correlated with a lower expression of CSE in the vessels of 129/Sv animals. It should also be noted that relaxation of aortic rings from C57BL/6J mice are even smaller (approximately 15%, data not shown). These observations taken together confirm that strain differences in H_2_S responses do exist, adding another level of complexity when comparing data from different studies.

Zhao et al [21] have previously shown that CSE, but not CBS, is expressed in the endothelium-free rat pulmonary artery, mesenteric artery, tail artery and aorta; they also proposed that CSE localizes to the smooth muscle cell layer of blood vessels. It later became apparent that cultured endothelial cells, as well as the endothelium in native vessels express CSE [13], [19], [44]. To determine the relative functional importance of each layer in H_2_S dilation, we tested the ability of endothelium-intact and endothelium-denuded aortic rings to relax to L-cysteine. Removal of the endothelium resulted in a significant decrease of L-cysteine-stimulated relaxation without affecting that ability of NaHS to dilate the vessels. On the other hand, we observed that in the mouse aorta, CSE is primarily expressed in the smooth muscle cell layer; however, lower CSE levels are present in the endothelium. The significant effect of endothelial denudation in L-cysteine dilation could be attributed to the fact that removal of the endothelial lining results in loss of NO production. Lack of NO is expected to inhibit H_2_S responses as the action of the two gasotransmitters on vascular tone and angiogenesis has been shown to be interdependent [45].

In summary, we have provided pharmacological and genetic evidence for the existence of a cGMP/PKG pathway downstream of H_2_S that regulates vascular tone. The two vasodilatory gasotransmitters, H_2_S and NO, regulate contractility by acting on the degradation and synthesis of cGMP, respectively. Convergence of the two pathways on the same effector (PKG) in the vessel wall, would allow for the fine-tuning of vascular tone, but also provide the redundancy needed to maintain vascular homeostasis and prevent disease development.

## Methods

### Ethics statement

All animal procedures were in compliance with the European Community guidelines for the use of experimental animals and approved by the Committee Centro Servizi Veterinari of the University of Naples “Federico II”; institutional regulations do not require the use of animal protocol numbers for approved protocols.

### Animals

Male and female mice CD-1 6 to 8 weeks old and 129/Sv were purchased from Harlan Laboratories (Italy). Mice carrying a null mutation of the gene encoding PKG-I (PKG-I−/− mice, also termed cGKI^L−/L−^ mice) and CSE−/− mice were generated as previously described [19], [46]. PKG-I−/− mice were on a 129/Sv genetic background and analyzed at an age of 10 to 16 weeks. Animals were housed in our animal facility having free access to water and food.

### Reagents

Cell culture media and serum were obtained from Life Technologies GIBCO-BRL (Paisley, UK). All cell culture plastic ware was purchased from Corning-Costar Inc. (Corning, NY). West Pico chemiluminescent substrate was purchased from Pierce Biotechnology (Rockford, Illinois); DC Protein assay kit, Tween 20 and other immunoblotting reagents were obtained from Bio-Rad Laboratories (Hercules, CA); penicillin and streptomycin were purchased from Applichem (Darmstadt, Germany). GYY4137, DT-2 and TAT were purchased from Cayman Chemical (Ann Arbor, Michigan), Biolog (Bremen, Germany) and Genscript (Piscataway, USA), respectively. The GAPDH, pVASP and secondary Abs were purchased from Cell Signaling Technologies (Beverly, MA), while the CSE antibody was obtained from Abnova Novus Biologicals (Littelton, CO). The PKG-I Ab used has been generated as previously described [47]. The anti-von Willebrand factor was obtained from Dako (Glostrup, Denmark), and the secondary anti-mouse and anti-rabbit antibodies used for the immunofluorescence studies were obtained from Life Technologies (Darmstadt, Germany). The cGMP EIA kit was obtained from Assay Designs (Ann Arbor, MI). Sildenafil, sodium hydrosulfide (NaHS), L-cysteine (L-cys), S-nitroso-N-acetylpenicillamine (SNAP), phenylephrine, protease/phosphatase inhibitors and all other chemicals used in solutions and buffers were purchased from Sigma Chemical Co (Milan, Italy). All drugs were dissolved in distilled water.

### *Ex vivo* studies

Animals were sacrificed with CO_2_ and thoracic aortas were rapidly harvested, dissected, and cleaned of adherent connective and fat tissue. Rings of about 1 mm length were denuded of the endothelium, cut and placed in organ baths (2.5 ml) filled with oxygenated (95% O_2_ -5% CO_2_) Krebs solution maintained at 37°C. The rings were connected to an isometric transducer (type 7006, Ugo Basile, Comerio, Italy) and changes in tension were recorded continuously with a computerized system (Data Capsule 17400, Ugo Basile, Comerio, Italy). Exclusively in the set of experiments performed on aortic rings harvested from PKG−/− and their respective background 129/Sv the endothelium was preserved. The composition of the Krebs solution was as follow (mM): NaCl 118, KCl 4.7, MgCl_2_ 1.2, KH_2_PO4 1.2, CaCl_2_ 2.5, NaHCO_3_ 25, and glucose 10.1. The rings were stretched until a resting tension of 1.5 g was reached and allowed to equilibrate for at least 45 min, during which time tension was adjusted, as necessary, to 1.5 g and bathing solution was periodically changed. In each experiment, rings were first challenged with PE (1 µM) until the responses were reproducible. The rings were then washed and contracted with PE (1 µM) and, once a plateau was reached, a cumulative concentration-response curve of the following drugs was performed: SNAP (100 pM–3 µM); NaHS (1 µM–300 mM); L-cys (1 µM–300 mM); GYY4137 (1 µM–300 µM). Rings were treated with the PKG inhibitor DT-2 or its control peptide TAT (1–3 µM; 20 minutes), or with PDE5 inhibitor sildenafil (1 nM; 15 minutes). After incubation time, cumulative concentration-response curve to SNAP; NaHS; L-cys; GYY4137 were performed. A preliminary study on the optimal incubation time and concentration of the drug treatments was carried out (data not shown). In another set of experiments, a cumulative concentration-response of NaHS and L-cys were carried out on aortic rings from PKG-I −/− and 129/Sv strains.

### Conscious systemic blood pressure measurement

Systolic blood pressure (SBP) was measured in conscious mice using the pneumatic tail-cuff method (W+W Blood pressure reporter, model 8006, Ugo Basile). Before the measurement, animals were preheated in a room at 30°C for 30 min, then they were placed in a plastic chamber. A cuff with a pneumatic pulse sensor was attached to the tail. This procedure was performed every day for 1 week before starting the experiments in order to habituate the animals to this procedure. During the entire measurement period, the temperature was maintained at 30°C. Two consecutive measurements were always recorded. SBP was measured and, once basal SBP was assessed, intraperitoneal injection of (D)-DT2 (100 nmoles) was performed. A more stable form of DT-2 [(D)-DT-2] that is composed of D-aminoacids was chosen for the *in vivo* experiments. SBP was then evaluated twice every 5 minutes. Fifteen minutes after the (D)-DT2 injection, NaHS (1 µmol/kg) was administered subcutaneously. SBP was then monitored every 5 minutes for three times. (D)-DT2 volume injected was 50 µl i.p., NaHS volume injected was 30 µl s.c. Both drugs were dissolved in saline.

### Cell culture

Rat aortic smooth muscle cells (RASMCs) were isolated from 12- to 14-wk-old male Wistar rats, five rats per isolation, as previously described [48]. Animals were anesthetized with pentobarbital sodium (40 mg/kg ip). Once fully anesthetized as judged by the lack of reaction to a noxious stimulus, animals were exsanguinated; thoracic aortas were then removed. More than 95% of cells isolated stained positive for smooth muscle α-actin. Cells between passages 2 and 5 were used for all experiments. RASMCs were routinely cultured in DMEM containing 4.5 g/l glucose and supplemented with 10% FBS and antibiotics.

### Western Blotting

Aortic tissues of CD-1 and 129/Sv were homogenized in modified RIPA buffer (Tris HCl 50 mM, pH 7.4, TritonX-100 1%, Sodium-deoxycholate 0.25%, NaCl 150 mM, EDTA 1 mM, phenylmethanesulphonylfluoride 1 mM, aprotinin 10 µg/ml, leupeptin 20 mM, NaF 50 mM) using a polytron homogenizer (two cycles of 10 s at maximum speed). In experiments performed to determine the expression of CSE in wild-type and PKG-I−/− animals, aortas from three animals were pooled and then homogenized. After centrifugation of homogenates at 12000 r.p.m for 15 min, protein concentration was determined by Bradford assay using BSA as standard. 40 µg of the denatured proteins were separated on 10% SDS/PAGE and transferred to a PVDF membrane. Membranes were blocked in PBS-Tween 20 (0.1%, v/v) containing 3% non fat dry milk for 1 hour at room temperature, and then incubated with the primary antibody overnight at 4°C. The filters were washed with PBS-tween 20 (0.1%, v/v) extensively for 30 min, before incubation, for 2 hours at 4°C, with the secondary antibody (1∶5000) conjugated with horseradish peroxidase anti-mouse IgG. The membranes were then washed and immunoreactive bands were visualized using a chemiluminescence substrate.

### cGMP measurements

Rat aortic smooth muscle cells were incubated for 5 min with the indicated concentration of the H_2_S donors. After the treatment, cells were washed with Hanks' balanced salt solution and cGMP was extracted using 0.1 N HCl. cGMP content was measured in the extracts using a commercially available enzyme immunoassay kit following the manufacturer's instructions.

For cGMP measurements in tissue and plasma of CSE knockout mice (CSE−/−), eight-week male CSE−/− and wild-type mice (CSE+/+) were used in this experiments. Animals were anesthetized with pentobarbital sodium (40 mg/kg ip) and exsanguinated. Blood plasma was prepared by spinning a tube of fresh blood containing EDTA (1500× g for 15 min at 4°C). The aorta and mesenteric artery tissues were dissected and cleaned for immediate cGMP measurement. First, aortic rings were placed in Krebs solution at 37°C and incubated for 30 min. After that, rings were stimulated with sodium nitroprusside (10 µM) for 2 min and then tissues were rapidly blotted weighted and quick frozen in liquid nitrogen. Tissues were snap frozen and homogenized in 1–3 volumes of buffer (containing 5% trichloroacetic acid) per gram of tissue and centrifuged at 1,500× g for 10 min. The supernatant was carefully removed and used in the next step. Residual TCA acid was removed by extraction into five volumes of water-saturated diethyl ether (repeated twice for a total of 3 extractions). Any residual ether was removed by warming the samples at 70°C for 5 min. The samples were then processed according to the instructions provided with commercially available enzyme immunoassay kit following the manufacture's instruction.

### Fluorescence Immunohistochemistry

To preserve tissue morphology and retain the antigenicity of the target molecules tissues were fixed in 4% paraformaldehyde in PBS for 1 hour and embedded in paraffin blocks. Subsequently, tissue sectioning was performed using a rotate microtome (5 µm thick) and sections were mounted on gelatin-coated histological slides. For the immunohistochemistry protocol, sections were rehydrated by immersion in xylene for 30 minutes, followed by immersion of slides in 100% ethanol for 3 minutes and sequentially in 95% ethanol, 70% ethanol, 50% ethanol for 2 minutes. The slides are rinsed with deionized H_2_O, rehydrated in PBS and incubated in proteinase K (20 µg/ml) in proteinase K buffer (100 mM Tris-HCl, pH 8.0; 50 mM EDTA) for 10 min at RT. After thorough washing with PBS, blocking of non-specific staining was performed, by incubation in blocking buffer (10% normal goat serum in PBS) for 60 minutes at RT, followed by application of primary antibodies (1∶200 rabbit polyclonal anti-von Willebrand Factor, Dako; 1∶500 anti-CSE diluted in blocking buffer overnight at 4°C. Slides are washed 3 times for 15 minutes each in PBS and incubated with secondary antibodies (1∶400 anti-mouse Alexa 488; 1∶400 anti-rabbit Alexa 568) for 2 hours at RT. Then, slides are washed 3 times for 15 minutes each and mounted using mounting medium with DAPI and coverslips. The staining was visualized using a confocal microscope with a digital camera attached (Leica) at a 200× magnification.

### Statistical analysis

Data were expressed as mean ± s.e.m. Statistical analysis was determined by using one or two way ANOVA and Dunnett's or Bonferroni as a post-test or t-test analysis when appropriate. Differences were considered statistically significant when P-value was less than 0.05. GraphPad Prism software (version 4.02, GraphPad Software, San Diego, CA) was used for all the statistical analysis.
